# High-Dose Intravenous Vitamin C in Critical Illness: A Translational Exposure–Response Framework for Biomarker-Guided Precision Therapy

**DOI:** 10.3390/medsci14030400

**Published:** 2026-07-17

**Authors:** Dejana Bajić, Ljiljana Andrijević, Nemanja Todorović, Mladena Lalić-Popović, Bojan Zarić, Stefan Dimić, Jelena Stojčević Maletić, Dajana Lendak, Boris Milijašević, Nataša Milošević

**Affiliations:** 1 Department of Biochemistry, Faculty of Medicine, University of Novi Sad, 21000 Novi Sad, Serbia; dejana.bajic@mf.uns.ac.rs (D.B.); ljiljana.andrijevic@mf.uns.ac.rs (L.A.); 015410@mf.uns.ac.rs (S.D.); jelena.stojcevic-maletic@mf.uns.ac.rs (J.S.M.); 2 Department of Pharmacy, Faculty of Medicine, University of Novi Sad, 21000 Novi Sad, Serbia; mladena.lalic-popovic@mf.uns.ac.rs (M.L.-P.); natasa.milosevic@mf.uns.ac.rs (N.M.); 3 Centre for Medical and Pharmaceutical Investigations and Quality Control (CEMPhIC), Faculty of Medicine, University of Novi Sad, 21000 Novi Sad, Serbia; 4 Institute for Pulmonary Diseases of Vojvodina, 21204 Sremska Kamenica, Serbia; 5 Department of Internal Medicine, Faculty of Medicine, University of Novi Sad, 21000 Novi Sad, Serbia; 6 Department of Infectious Diseases, Faculty of Medicine, University of Novi Sad, 21000 Novi Sad, Serbia; 7 Clinic for Infectious Diseases, University Clinical Center of Vojvodina, 21000 Novi Sad, Serbia; 8 Department of Clinical Pharmacology and Toxicology, Faculty of Medicine, University of Novi Sad, 21000 Novi Sad, Serbia

**Keywords:** ascorbic acid, critical illness, COVID-19, sepsis, biomarkers, precision medicine, pharmacokinetics, pharmacodynamics

## Abstract

High-dose intravenous vitamin C (HDIVC) has been investigated as a potential adjunctive therapy in critical illness, including sepsis, acute respiratory distress syndrome (ARDS), and COVID-19. Despite a strong mechanistic rationale, clinical trials have yielded inconsistent results. From a clinical pharmacology perspective, this variability may reflect, at least in part, differences in pharmacokinetic exposure, timing of administration, and patient selection rather than a lack of biological activity. Intravenous administration enables plasma concentrations in the millimolar range (≈1–5 mM), far exceeding those achievable with oral dosing (<100 µM), thereby reaching thresholds required for pharmacodynamic effects on oxidative stress, immune signaling, and endothelial function. This exposure-dependent transition distinguishes vitamin C as a pharmacological agent rather than a nutritional supplement in critically ill populations. Therapeutic response may be influenced by timing relative to disease progression, with earlier administration representing a biologically plausible strategy that warrants prospective evaluation rather than a clinically established therapeutic window. Interindividual variability in transporter function, redox status, and genetic background may further contribute to heterogeneous responses. Biomarkers such as interleukin-6 (IL-6), C-reactive protein (CRP), D-dimer, and markers of endothelial injury provide a framework for patient stratification and monitoring of pharmacodynamic effects. Integrated with pharmacokinetic principles, these markers support a shift toward biomarker-guided, precision-based therapeutic strategies. This review synthesizes current clinical and mechanistic evidence through an exposure–response conceptual framework, framing HDIVC as a context-dependent pharmacological intervention and advancing a shift toward biomarker-guided, precision-based therapeutic strategies in critical illness.

## 1. Introduction

Despite extensive investigation, the clinical efficacy of high-dose intravenous vitamin C in critical illness remains uncertain, reflecting a fundamental gap between mechanistic rationale and clinical outcomes. This redox–immune–endothelial triad provides a mechanistic basis for targeted therapeutic intervention rather than uniform treatment approaches [1,2,3,4].

Unlike oral supplementation, whose systemic exposure is constrained by saturable intestinal absorption and homeostatic renal regulation, intravenous administration produces pharmacological plasma concentrations that are unattainable by the oral route. Consequently, intravenous vitamin C exhibits pharmacodynamic properties that extend beyond correction of nutritional deficiency, including modulation of redox signaling, inflammatory pathways, endothelial function, and cellular bioenergetics [5,6].

Interest in the therapeutic potential of vitamin C was largely stimulated by the pioneering work of Linus Pauling and Ewan Cameron, who proposed that pharmacological doses of vitamin C might prolong survival and improve quality of life in patients with advanced cancer [7]. Although their early clinical observations generated considerable scientific interest, subsequent randomized trials using exclusively oral vitamin C failed to reproduce these benefits [5,8,9]. It is now recognized that these apparently conflicting findings were largely attributable to fundamental pharmacokinetic differences between oral and intravenous administration. Oral supplementation is tightly regulated by saturable intestinal absorption and renal excretion, resulting in plasma concentrations that rarely exceed physiological micromolar levels. In contrast, intravenous administration bypasses intestinal transport limitations and achieves transient supraphysiological millimolar plasma concentrations capable of modulating redox-sensitive signaling pathways, endothelial function, and inflammatory responses [5,6]. This paradigm shift transformed vitamin C from a nutritional supplement into a pharmacological agent whose biological activity depends on route of administration, systemic exposure, and disease-specific biological context [6].

Despite this rationale, clinical trials of high-dose intravenous vitamin C have yielded inconsistent results [4,10,11]. This variability may, at least in part, reflect differences in patient selection, timing of administration, and achieved pharmacokinetic exposure, rather than a uniform lack of biological activity [6,12]. From a clinical pharmacology perspective, this variability can be interpreted through an exposure–response framework, where therapeutic effect is contingent on achieving plasma concentrations sufficient to modulate redox-sensitive pathways. Available pharmacokinetic data indicate that intravenous administration can increase circulating ascorbate levels from physiological micromolar ranges (<100 μM) to supraphysiological millimolar concentrations (>1–5 mM), a threshold at which pharmacodynamic effects on oxidative stress and inflammatory signaling become biologically relevant [5,6].

Within this context, the concept of precision redox medicine may be viewed as a translational conceptual framework that may inform future biomarker-guided clinical investigation rather than a clinically established precision-treatment strategy [12,13]. Biomarkers such as IL-6, CRP, and D-dimer may therefore serve not only as markers of disease severity but also may provide a biologically plausible basis for future biomarker-guided therapeutic stratification, although their predictive utility remains to be prospectively established [4,13].

This review examines intravenous vitamin C from a clinical pharmacology perspective, integrating pharmacokinetic considerations with biomarker-guided precision therapy concepts. By aligning treatment with patient-specific redox, immune, and endothelial profiles, this approach aims to move from empirical use toward targeted, phenotype-driven therapy in critical illness [13]. Addressing this gap, the present review integrates pharmacokinetic exposure with biomarker-defined phenotypes and proposes a translational framework intended to inform future biomarker-guided precision pharmacotherapy research [12].

### Methods of Literature Search and Study Selection

A comprehensive structured narrative review was conducted to evaluate the clinical pharmacology, mechanistic rationale, and therapeutic role of high-dose intravenous vitamin C in critically ill adult patients. The review was designed to provide a comprehensive and critical synthesis of both clinical and mechanistic evidence relevant to biomarker-guided precision pharmacotherapy, rather than to address a single predefined research question through formal systematic review methodology. For the purpose of this review, critical illness refers to acute life-threatening conditions requiring intensive care management and characterized by actual or imminent organ dysfunction resulting from dysregulated inflammatory, immune, and redox responses, including sepsis, septic shock, acute respiratory distress syndrome (ARDS), and severe COVID-19 [14,15].

A structured literature search was performed in PubMed and Web of Science Core Collection from database inception through June 2026. During manuscript revision, the search was updated to identify newly published peer-reviewed studies, systematic reviews, meta-analyses, and evidence-based clinical practice guidelines relevant to intravenous vitamin C in critical illness. To minimize the possibility of missing relevant publications, an additional manual search was conducted using Google Scholar and the reference lists of selected articles.

To comprehensively address the objectives of this review, two complementary literature search strategies were employed. The first strategy focused on identifying clinical evidence evaluating the efficacy and safety of high-dose intravenous vitamin C in critically ill adults, including randomized controlled trials, observational studies, systematic reviews, meta-analyses, and international clinical practice guidelines. The second strategy focused on mechanistic and translational evidence addressing pharmacokinetics, pharmacodynamics, exposure–response relationships, redox biology, endothelial dysfunction, inflammatory signaling, biomarker-guided therapy, and related molecular mechanisms relevant to precision pharmacotherapy. Search strategies were adapted to the syntax of each database using combinations of controlled vocabulary and free-text terms linked by Boolean operators (“AND”, “OR”).

The primary evidence base included publications from 2004 through June 2026, encompassing randomized controlled trials, observational studies, mechanistic investigations, systematic reviews, meta-analyses, and international evidence-based clinical practice guidelines. The year 2004 was selected because the landmark pharmacokinetic study by Padayatty et al. fundamentally established the distinction between oral and intravenous vitamin C administration and provided the scientific basis for subsequent translational and clinical investigations. Earlier landmark publications were included selectively to provide the historical background and scientific context of the evolution of intravenous vitamin C therapy. Studies focusing exclusively on oral vitamin C supplementation, non-peer-reviewed publications, conference abstracts without full-text articles, editorials lacking original scientific content, and studies unrelated to critical illness were excluded.

Preference was given to high-quality peer-reviewed publications, contemporary systematic reviews and meta-analyses, international evidence-based clinical practice guidelines, and landmark mechanistic studies whenever available. Clinical and mechanistic evidence were synthesized narratively within a redox–immune–endothelial framework to identify biologically plausible exposure–response relationships and clinically relevant biomarker-guided therapeutic strategies. Rather than providing a purely descriptive summary, the evidence synthesis was designed to support the development of a translational exposure–response framework for precision pharmacotherapy with high-dose intravenous vitamin C.

## 2. Pharmacological Rationale of Vitamin C

### 2.1. Redox-Modifying Function of Vitamin C

Vitamin C (ascorbic acid) functions as a redox-active molecule that modulates cellular responses to oxidative stress rather than acting solely as a passive antioxidant. It directly neutralizes reactive oxygen and nitrogen species (ROS/RNS), including superoxide, hydrogen peroxide, hydroxyl radicals, nitric oxide, and peroxynitrite, thereby limiting oxidative damage to lipids, proteins, and DNA [16,17,18,19]. At the same time, it supports antioxidant networks by regenerating molecules such as vitamin E and glutathione, sustaining intracellular redox balance [16,17,18,19,20].

In critical illness, this balance is disrupted. Sepsis and severe COVID-19 are characterized by excessive ROS/RNS production driven by mitochondrial dysfunction and hyperinflammation, leading to endothelial injury, impaired cellular function, and organ dysfunction [20,21]. These processes reinforce each other, amplifying inflammatory signaling and microvascular damage.

Within this context, the effects of intravenous vitamin C can be interpreted across three interconnected domains: reduction of oxidative stress, modulation of immune responses, and stabilization of endothelial function. Clinically, this suggests that therapeutic benefit may depend on targeting patients with evidence of redox imbalance and related downstream dysfunction, rather than uniform administration. Importantly, these redox-modulating effects are concentration-dependent, with significant scavenging of reactive species and modulation of redox-sensitive signaling pathways occurring predominantly at pharmacological plasma concentrations achievable only through intravenous administration.

### 2.2. Vitamin C Role in Neuroendocrine Homeostasis

**Figure 1:**
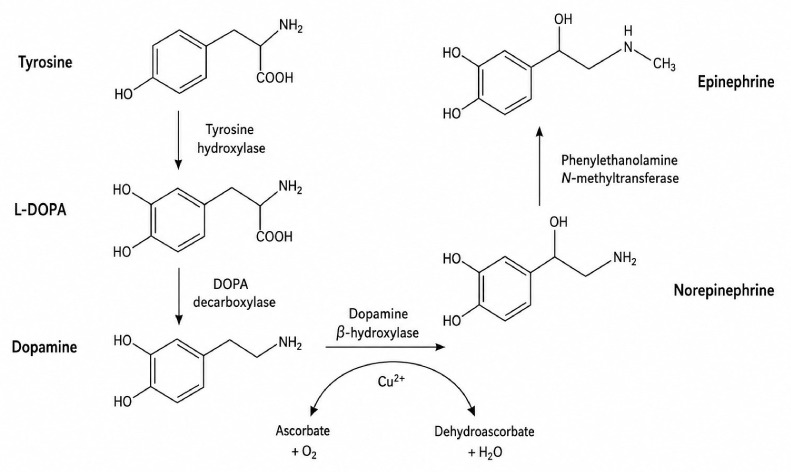
Catecholamine biosynthesis pathway highlighting the role of vitamin C as a cofactor for dopamine β-hydroxylase in the conversion of dopamine to norepinephrine, a key step relevant to vascular tone regulation in critical illness.

Vitamin C plays a direct role in neuroendocrine regulation through its function as a cofactor for dopamine β-hydroxylase (Figure 1), the enzyme responsible for converting dopamine to norepinephrine in adrenal and sympathetic tissues [22].

During critical illness, increased catecholamine demand often exceeds endogenous synthesis, contributing to vasopressor dependence. In this context, intracellular vitamin C depletion may impair norepinephrine production, potentially exacerbating hemodynamic instability [22,23].

This mechanism is clinically relevant in septic patients, where vasopressor requirements reflect the severity of circulatory dysfunction. Intravenous vitamin C has therefore been proposed as an adjunctive strategy to support endogenous catecholamine synthesis and improve vascular responsiveness under conditions of severe physiological stress [22,23,24].

### 2.3. Vitamin C Immunomodulatory Potential

Severe conditions such as ARDS, sepsis, and COVID-19 are characterized by dysregulated immune responses driven, in part, by activation of inflammasomes, particularly NLRP3. This process promotes the release of pro-inflammatory cytokines, including IL-1β and IL-18, and induces pyroptosis, contributing to tissue injury and systemic inflammation [25,26].

Preclinical evidence suggests that vitamin C may modulate these pathways by reducing reactive oxygen species (ROS), which act as upstream signals for inflammasome activation. This redox-dependent effect provides a plausible explanation for observed reductions in inflammatory markers in some clinical settings following high-dose intravenous administration [25,26,27].

In parallel, the interaction between the SARS-CoV-2 spike glycoprotein and the ACE2 receptor is critically influenced by extracellular thiol–disulfide homeostasis. Structural integrity of the receptor-binding domain depends on conserved disulfide bonds, whereas changes in the redox environment may alter the accessibility and affinity of spike–ACE2 binding [28,29]. Experimental, computational, and biochemical studies further suggest that oxidative modification of critical cysteine residues within the spike protein or ACE2 receptor may reduce receptor-binding affinity, supporting a biologically plausible redox-sensitive mechanism influencing viral entry. However, the clinical significance of this mechanism remains to be confirmed in vivo [5,30,31].

Taken together, these findings indicate that vitamin C may influence immune responses through redox-mediated modulation of inflammatory signaling and, potentially, redox-dependent host–virus protein interactions. Clinically, this supports further investigation in biomarker-defined patient subsets, while current evidence remains insufficient for definitive therapeutic conclusions [5,21,30,31].

## 3. Pharmacokinetics and Pharmacodynamics of Intravenous Vitamin C Administration

From a clinical pharmacology perspective, the pharmacodynamic activity of vitamin C depends on achieving pharmacologically relevant plasma concentrations, which are primarily determined by the route of administration. In this context, exposure rather than nominal dose becomes the key determinant of pharmacodynamic response. Oral dosing is limited by saturable intestinal transport, whereas intravenous administration bypasses these constraints and enables substantially higher systemic exposure, allowing biologically meaningful modulation of redox, immune, and endothelial pathways [5,32,33] (Table 1).

This distinction is particularly relevant in critical illness, where oxidative stress and inflammatory activation increase vitamin C turnover and depletion. Consequently, intravenous dosing may be required not only to restore baseline levels but also to achieve concentrations at which pharmacodynamic effects become clinically relevant [5,11,32,33]. Plasma concentrations in the millimolar range (≈1–5 mM), achievable only through intravenous administration, represent a pharmacodynamic threshold at which vitamin C transitions from a nutritional antioxidant to a pharmacological agent capable of modulating cellular signaling, endothelial function, and inflammatory cascades. Although the optimal dosing strategy has not been established, intermittent high-dose infusions may generate higher peak concentrations, whereas continuous infusion may provide more stable exposure, although the optimal strategy remains to be defined.

The temporal profile of exposure may further influence therapeutic outcomes. Intermittent administration produces transient peak concentrations that may enhance redox signaling, while continuous infusion maintains sustained exposure that may be more relevant for prolonged modulation of endothelial and inflammatory pathways. Interindividual variability in renal clearance and volume of distribution may further influence achieved exposure, particularly in critically ill patients with altered organ function.

Following infusion, vitamin C is rapidly distributed and taken up into cells via sodium-dependent vitamin C transporters (SVCT1/2). Inflammatory states may alter transporter expression and function, introducing variability in intracellular exposure and potentially contributing to heterogeneous clinical responses [32]. At high plasma concentrations, transporter-mediated cellular uptake may become a limiting step, introducing a potential disconnect between systemic exposure and intracellular pharmacodynamic effect, particularly in inflamed tissues where transporter expression may be altered.

Emerging clinical data provide partial support for these mechanisms. In post hoc analyses of the CITRIS-ALI trial, intravenous vitamin C was associated with reductions in biomarkers of endothelial injury, including syndecan-1 and circulating cell-free DNA, alongside improvements in oxygenation indices, suggesting effects on vascular integrity and pulmonary function [34].

Intravenous vitamin C is generally well tolerated, although safety considerations include oxalate nephropathy in renal impairment, hemolysis in G6PD deficiency, and interference with point-of-care glucose measurements [35,36].

Taken together, these findings indicate a clear exposure-dependent pharmacodynamic profile, in which dose, timing, and patient-specific factors determine therapeutic effect. These findings support further investigation of biomarker-guided precision treatment strategies rather than their current implementation in routine clinical practice [5,11,32,33,34,35,36].

## 4. Clinical Evidence and Therapeutic Potential of Intravenous Vitamin C in COVID-19, Sepsis and ARDS

Clinical evidence on high-dose intravenous vitamin C (HDIVC) in critical illness remains heterogeneous; however, when interpreted within a redox–immune–endothelial framework, consistent patterns emerge. A structured synthesis of key trials, dosing strategies, and outcomes is provided in Table 2, highlighting variability across populations and study designs. Overall, randomized trials and meta-analyses have not demonstrated a consistent mortality benefit. However, mechanistic studies, pharmacokinetic data, and emerging clinical observations suggest that differences in pharmacological exposure, timing of administration, and biological phenotype may contribute to the observed heterogeneity of clinical outcomes. An alternative interpretation is that high-dose intravenous vitamin C may have limited or no clinically meaningful efficacy in some or all critically ill populations despite measurable biological activity. The currently available evidence does not allow these competing explanations to be distinguished with certainty [10,11,37,38,39,40,41,42].

Although this hypothesis has not yet been prospectively validated, it provides a biologically plausible framework for interpreting apparently discordant clinical findings and for designing future biomarker-guided clinical trials. However, reproducible signals of biological activity—including reductions in inflammatory markers (IL-6, CRP), decreased vasopressor requirements, and improved oxygenation—are observed across multiple studies [11,13,37,38,39,41,42,43,44,45,46,47,48,49,50,51].

Importantly, the currently available clinical evidence does not establish a direct causal relationship between pharmacokinetic exposure, timing of administration, patient phenotype, and clinical outcome. Rather, the exposure–response framework proposed in this review represents a biologically plausible and pharmacologically grounded interpretative model that integrates mechanistic evidence, pharmacokinetic principles, and emerging clinical observations. Future prospective trials incorporating pharmacokinetic monitoring together with biomarker-defined patient stratification will be required to validate this hypothesis [12].

Recent evidence published during manuscript revision further reinforces the concept that the clinical efficacy of high-dose intravenous vitamin C depends primarily on patient selection, pharmacological exposure, timing of administration, and underlying biological phenotype rather than universal treatment effects [11,12]. Although recent systematic reviews continue to demonstrate modest improvements in physiological and organ function–related outcomes, consistent reductions in mortality have not been established across heterogeneous critically ill populations [10,42]. Collectively, these findings support further investigation of competing hypotheses, including both the possibility that clinical benefit depends on appropriate pharmacological exposure and patient selection, and the alternative possibility that HDIVC has limited efficacy across heterogeneous critically ill populations [11,12]. Future adequately powered randomized controlled trials integrating pharmacokinetic monitoring and validated biomarkers are required to identify patients most likely to benefit from HDIVC therapy [10,20].

In COVID-19, a 2024 meta-analysis of over 1,500 patients found no significant reduction in in-hospital mortality, although trends toward shorter ICU stay and reduced mechanical ventilation were noted [13]. Similarly, the LOVIT-COVID trial showed no difference in organ support–free days, yet post hoc analyses suggested reductions in IL-6, CRP, and vasopressor use with early administration [41]. Other studies [38,39,42] reported improvements in surrogate endpoints such as PaO_2_/FiO_2_ and SOFA scores without consistent survival benefit, underscoring the influence of timing, dosing, and baseline severity [38,39,42]. These observations raise the possibility of a therapeutic window in which intervention occurs at a stage when redox imbalance and endothelial dysfunction remain pharmacologically modifiable. Delayed administration, even at adequate doses, may fail to produce clinical benefit if downstream injury pathways have already become irreversible.

In sepsis and ARDS, findings remain similarly variable. Trials such as CITRIS-ALI and subsequent studies report improvements in oxygenation, organ dysfunction scores, and ICU-related outcomes, but without consistent mortality reduction [11,38,43,46,47,48,49,50,51]. An equally important interpretation is that the absence of consistent clinical benefit across randomized trials may reflect limited therapeutic efficacy of HDIVC in unselected critically ill populations These discrepancies may, at least in part, be explained by differences in pharmacokinetic exposure and the timing of therapy relative to the evolving pathophysiology of critical illness. From a pharmacological perspective, inadequate achievement and maintenance of pharmacologically active plasma vitamin C concentrations early in the disease course could plausibly attenuate potential therapeutic effects. Likewise, suboptimal dosing regimens used in some clinical trials may have contributed to insufficient systemic exposure, although this hypothesis has not yet been prospectively validated. Accordingly, the exposure–response framework proposed in this review should be regarded as a biologically plausible interpretative model integrating pharmacokinetic, mechanistic, and emerging clinical evidence rather than as definitive proof of causality. Future prospective studies incorporating pharmacokinetic monitoring together with biomarker-guided patient stratification will be required to validate this hypothesis.

Adjunctive redox-targeted strategies provide additional support for this interpretation. N-acetylcysteine enhances glutathione synthesis and ROS scavenging, with clinical data suggesting improved oxygenation but limited by tolerability at higher doses [52,53,54]. Combination approaches with vitamin C may augment intracellular redox buffering and improve resilience to oxidative injury [52,53,54,55,56,57]. Melatonin, through mitochondrial protection and NLRP3 modulation, represents another complementary agent with emerging translational relevance [55,58]. Additional combinations, including vitamin D and zinc, aim to reinforce antioxidant and immune pathways, although robust clinical validation is still lacking [59,60,61]. The Marik protocol illustrates this multi-targeted approach, with observational benefits not consistently reproduced in randomized trials [36].

Taken together, available evidence suggests that HDIVC exerts measurable pharmacodynamic effects, while clinical efficacy remains context-dependent. The heterogeneity of outcomes likely reflects variability in exposure (dose and administration strategy), timing relative to disease progression, and patient phenotype.

From a clinical pharmacology perspective, high-dose intravenous vitamin C should not be considered a universal intervention for all critically ill patients, but rather an exposure-dependent therapy whose effects are contingent on achieving adequate plasma concentrations at a stage when redox imbalance, immune dysregulation, and endothelial injury remain modifiable.

Across studies, variability in outcomes appears closely linked to differences in timing of administration, dosing strategies, and patient selection. Earlier initiation of therapy, particularly during the initial phase of critical illness before irreversible tissue injury becomes established, together with achievement of pharmacologically active plasma concentrations and appropriate patient selection, may represent important determinants of therapeutic response. Although several studies suggest potential benefits of earlier administration, the currently available evidence does not support defining a specific therapeutic window, and this concept should be regarded as a biologically plausible hypothesis requiring prospective validation.

In this context, intravenous vitamin C, alone or in combination with agents such as N-acetylcysteine or melatonin, may be most appropriately positioned as part of a biomarker-guided, precision-based therapeutic strategy rather than routine supplementation in unselected populations [11,13,38,43,46,47,48,49,50,51,56,57,58,59,60,61,62]. Collectively, these findings are consistent with an exposure–response interpretation, although alternative explanations, including intrinsically limited clinical efficacy across heterogeneous critically ill populations, remain equally plausible on the basis of current evidence.

Viewed through this conceptual framework, an important question shifts from whether high-dose intravenous vitamin C is universally effective to under which pharmacological and biological conditions it may exert clinically meaningful effects. Although this hypothesis remains to be prospectively validated, it provides a biologically plausible translational framework for future biomarker-guided clinical investigations and precision pharmacotherapy.

## 5. Key Factors Modulating the Clinical Impact of IV Vitamin C Therapy

Within the framework of precision redox medicine, variability in therapeutic response to intravenous vitamin C reflects the interplay between pharmacological exposure and individual biological context. Two principal determinants emerge: formulation and dosing characteristics (summarized in Table 3) and interindividual genetic variability affecting transport, distribution, and redox metabolism (Table 4).

From a pharmacological perspective, the clinical effect of IV vitamin C is not defined solely by dose, but by the ability to achieve and maintain effective plasma and intracellular concentrations. Recent population pharmacokinetic data further demonstrated substantial interindividual variability in vitamin C exposure despite standardized dosing, highlighting renal function and illness severity as important determinants of pharmacokinetic variability [12]. Differences in formulation (ascorbic acid vs. sodium ascorbate), dosing schedules, and infusion strategies directly influence pharmacokinetic exposure and, consequently, pharmacodynamic response. For example, Fowler et al. demonstrated that repeated dosing (50 mg/kg every 6 h) was associated with reductions in organ dysfunction and inflammatory markers in sepsis and ARDS [11], while Zhang et al. reported improved oxygenation and ICU outcomes with 12 g/day in critically ill COVID-19 patients [38]. Combination regimens, including thiamine and corticosteroids, further suggest that modulation of interconnected metabolic and inflammatory pathways may enhance clinical effects [11,38]. These observations underscore that dosing strategy and formulation are not interchangeable variables but important determinants of pharmacokinetic exposure and pharmacodynamic activity, both of which may influence therapeutic response.

At the same time, genetic variability introduces an additional layer of complexity. Polymorphisms in vitamin C transporters (SLC23A1/SLC23A2) and redox-related enzymes (GSTM1, G6PD) may influence intracellular vitamin C availability, antioxidant capacity, and safety profile [59,60,61]. In addition to genetic variation, differences in transporter expression and regulation may further contribute to interindividual variability in tissue vitamin C distribution and pharmacological response [67,68,69,70]. Altered transporter function may limit tissue uptake, GSTM1 deficiency may impair glutathione recycling, and G6PD deficiency increases susceptibility to oxidative hemolysis under high-dose exposure [71,72,73,74]. Given the central role of glutathione in cellular antioxidant defense, GST polymorphisms may modify the biological response to pharmacological vitamin C exposure; however, current evidence remains largely indirect and observational [65,70]. Experimental studies further suggest that endothelial vitamin C transport is tightly regulated and may become particularly relevant under conditions of oxidative stress and critical illness, although its clinical implications during intravenous therapy remain incompletely understood [69].

For clinicians, pharmacologists, and laboratory specialists alike, these findings converge on a central principle: therapeutic response is shaped not only by the drug, but by the biological system receiving it. Integrating dosing strategy with pharmacogenetic and metabolic context supports a shift toward biomarker-informed, genotype-aware treatment algorithms, aligning IV vitamin C therapy with the broader paradigm of precision pharmacotherapy [72,73]. Taken together, these observations support future investigation of pharmacogenetic stratification as one potential component of biomarker-informed precision pharmacotherapy rather than as a currently validated clinical decision-making tool [64].

Collectively, these observations support the hypothesis that pharmacogenetic and biological variability may contribute to interindividual differences in pharmacological response. However, prospective studies are required to determine whether these factors define clinically relevant responder and non-responder phenotypes. Patients with preserved transporter function, intact redox buffering capacity, and early-stage disease may be more likely to achieve sufficient intracellular exposure and demonstrate clinical benefit, whereas those with impaired transport, advanced oxidative injury, or genetic susceptibility may exhibit attenuated or adverse responses.

### Safety Considerations and Drug Interactions of High-Dose Intravenous Vitamin C

Although high-dose intravenous vitamin C (HDIVC) is generally considered to have a favorable safety profile when administered under appropriate clinical supervision, its pharmacological use requires careful consideration of patient-specific risk factors, organ function, and potential drug interactions [75]. As with any exposure-dependent therapy, the therapeutic window of HDIVC is influenced not only by dose and pharmacokinetics but also by the biological characteristics of the recipient. Consequently, patient selection and appropriate monitoring represent essential components of precision-based vitamin C therapy rather than secondary safety measures [76].

One of the best-characterized adverse events associated with HDIVC is secondary oxalate nephropathy. Vitamin C is metabolized to oxalate, and supraphysiological intravenous doses may increase urinary oxalate excretion. In patients with impaired renal function, reduced glomerular filtration, dehydration, or pre-existing chronic kidney disease, oxalate accumulation may promote calcium oxalate crystal deposition within renal tubules, potentially contributing to acute kidney injury [77]. Although this complication appears uncommon, reported cases emphasize the importance of renal function assessment before treatment initiation and continuous monitoring of serum creatinine, urine output, and renal recovery during therapy [75,76,77]. In patients requiring renal replacement therapy, oxalate can be effectively removed by dialysis, which may reduce systemic oxalate accumulation during treatment; however, continued monitoring remains necessary, particularly in the setting of ongoing oxalate production or severe renal impairment [78].

Another clinically relevant safety consideration is glucose-6-phosphate dehydrogenase (G6PD) deficiency. Because erythrocytes lacking adequate G6PD activity have reduced capacity to regenerate reduced glutathione through the pentose phosphate pathway, exposure to pharmacological concentrations of vitamin C may, under specific conditions, increase susceptibility to oxidative hemolysis. Because clinically significant hemolysis has been reported after high-dose exposure in G6PD-deficient individuals, screening should be considered before pharmacological-dose IV vitamin C, particularly when very high doses are planned [75,76,79,80,81]. Although clinically significant hemolysis has been reported following pharmacological-dose intravenous vitamin C administration in G6PD-deficient individuals, the available evidence is derived primarily from case reports and observational data. Therefore, screening should be considered before pharmacological-dose IV vitamin C, particularly when very high doses are planned [75,76,79,80,81]. This precaution exemplifies the growing importance of integrating pharmacogenetic information into individualized therapeutic decision-making. Current Clinical Pharmacogenetics Implementation Consortium (CPIC) guidance likewise emphasizes consideration of G6PD genotype when prescribing medications associated with oxidative stress. Although vitamin C is not classified as a high-risk medication, these recommendations further support individualized risk assessment and careful clinical monitoring when pharmacological-dose intravenous vitamin C is administered in susceptible patients [81].

Patients with hereditary hemochromatosis or clinically significant iron overload require particular caution during high-dose intravenous vitamin C therapy. Vitamin C promotes the reduction of ferric (Fe^3+^) to ferrous (Fe^2+^) iron, thereby increasing its redox activity and potentially amplifying iron-catalyzed oxidative injury through Fenton chemistry in the presence of excessive body iron stores [75,82]. Although clinically significant toxicity appears uncommon, prolonged pharmacological-dose vitamin C administration should be avoided or carefully monitored in patients with documented iron overload [75,76].

An additional practical consideration involves interference with point-of-care glucose monitoring systems [83,84]. Pharmacological plasma concentrations achieved after intravenous administration may produce falsely elevated glucose readings with certain electrochemical glucometers utilizing glucose dehydrogenase- or oxidase-based methodologies. Such analytical interference may result in inappropriate insulin administration and subsequent severe hypoglycemia [84,85]. Therefore, laboratory-based plasma glucose measurements should be preferred during and immediately after HDIVC infusion whenever accurate glycemic assessment is clinically required [8,84].

Potential drug interactions also warrant consideration. The antioxidant and redox-active properties of vitamin C have generated theoretical concerns regarding concomitant administration with selected chemotherapeutic agents whose cytotoxic activity may depend, at least in part, on reactive oxygen species generation [83,86]. These concerns should not be generalized across all anticancer regimens because available evidence suggests agent-specific and context-dependent effects rather than a universal antagonistic interaction [86,87]. For example, preclinical studies have suggested a potential antagonistic interaction between vitamin C and bortezomib, whereas clinical studies evaluating IV vitamin C in combination with gemcitabine-, platinum-, taxane-, or fluoropyrimidine-based regimens have generally demonstrated acceptable safety without consistent evidence of reduced antitumor efficacy [76,87]. Consequently, decisions regarding HDIVC administration in oncology patients should remain individualized and coordinated with the treating oncologist until additional prospective evidence becomes available [76,86].

Similarly, evidence regarding interactions between HDIVC and oral anticoagulants such as warfarin remains limited and inconclusive. Although isolated case reports have suggested possible alterations in anticoagulant response during high-dose vitamin C administration, clinically meaningful interactions have not been consistently confirmed in controlled studies [88]. Nevertheless, monitoring of the international normalized ratio (INR) is reasonable when HDIVC is initiated or discontinued in patients receiving long-term warfarin therapy [76,88].

Collectively, these safety considerations highlight that successful implementation of HDIVC extends beyond dose selection alone. Assessment of renal function, G6PD status, iron metabolism, concomitant medications, and analytical limitations of laboratory testing should be integrated into treatment algorithms [10,75]. Such an approach aligns safety monitoring with the same biomarker-guided and patient-centered framework that underpins therapeutic efficacy, reinforcing the concept that precision pharmacotherapy requires simultaneous optimization of both benefit and risk [75,76].

Thus, safety monitoring should be integrated into the same biomarker-guided precision medicine framework as efficacy monitoring, with renal function, G6PD status, iron metabolism, concomitant medications, and potential analytical interference systematically evaluated before and during HDIVC therapy [70,75,76].

## 6. Critical Biomarkers for Monitoring IV Vitamin C Efficacy

Biomarkers represent the operational interface between mechanism and clinical decision-making in precision redox medicine. As summarized in Table 5, markers of inflammation, oxidative stress, endothelial dysfunction, and coagulopathy provide complementary insights into disease trajectory and therapeutic response in critically ill patients [64,65,66,89].

Interleukin-6 (IL-6) reflects the intensity of systemic inflammatory activation and is consistently associated with disease severity and mortality [90,91]. Observed reductions in IL-6 following IV vitamin C administration suggest modulation of upstream inflammatory signaling rather than isolated cytokine suppression [92]. In parallel, C-reactive protein (CRP) offers a readily accessible marker of systemic inflammation, allowing dynamic monitoring of treatment response in clinical settings [93,94].

Oxidative stress biomarkers, including malondialdehyde (MDA) and F2-isoprostanes, capture the biochemical burden of lipid peroxidation and cellular injury [95,96]. Their reduction under vitamin C therapy reflects restoration of redox balance at the molecular level, linking pharmacodynamic effect with measurable biochemical change.

Endothelial markers, such as von Willebrand factor (vWF) and soluble thrombomodulin (sTM), provide insight into vascular injury and dysfunction, central components of critical illness pathophysiology [97,98]. Improvements in these parameters suggest stabilization of the endothelial barrier, a key therapeutic target in sepsis and ARDS.

D-dimer, as an indicator of coagulation activation and fibrinolysis, integrates inflammatory and endothelial signals, reflecting the thrombo-inflammatory axis characteristic of severe disease [99]. Decreases in D-dimer may indicate attenuation of inflammation-driven coagulopathy, although interpretation should remain context-dependent [100].

Taken together, these biomarkers do not function in isolation but define a multidimensional profile of disease biology. For clinicians, they guide patient selection and monitoring; for pharmacologists, they represent measurable pharmacodynamic endpoints; and for translational researchers, they provide a framework for linking molecular mechanisms with clinical outcomes. This integrated approach enables a shift from descriptive biomarker use toward actionable, phenotype-driven therapeutic strategies in critically ill populations [73,89,90,91,92,93]. From a pharmacodynamic perspective, these biomarkers may serve as intermediate endpoints reflecting target engagement and biological response to therapy.

While the biomarkers summarized in Table 5 primarily reflect therapeutic response and pharmacodynamic target engagement, successful implementation of high-dose intravenous vitamin C also requires systematic safety monitoring. Beyond evaluating biological efficacy, individualized patient management should incorporate assessment of treatment-related risks, including susceptibility to oxidative hemolysis, renal complications, analytical interference, and iron metabolism. The principal safety-monitoring parameters recommended before and during pharmacological-dose intravenous vitamin C therapy are summarized in Table 6.

## 7. From Mechanistic Insight to Translational Framework: Precision Redox-Immune-Endothelial Phenotyping in Critical Illness

Accumulating mechanistic and clinical evidence indicates that sepsis, ARDS, and severe COVID-19 are not uniform entities but heterogeneous syndromes shaped by interacting disturbances in redox balance, immune activation, and endothelial function. This biological variability likely underlies the limited success of unstratified therapeutic trials and supports a shift toward biomarker-guided strategies that align treatment with underlying pathophysiology [101].

At the molecular level, oxidative stress amplifies immune and endothelial dysfunction through interconnected signaling pathways. Biomarkers of lipid peroxidation (e.g., malondialdehyde), inflammation (IL-6, CRP), and endothelial injury (D-dimer, soluble thrombomodulin, vWF) consistently correlate with disease severity and outcomes [91,92,93,94,95,96,97,98,99,102,103]. When integrated, these markers define multidimensional phenotypes that may ultimately support predictive enrichment strategies once prospectively validated —identifying candidate patient subgroups for future evaluation of targeted interventions [102,103] (Table 7).

At present, these biomarkers should be regarded primarily as indicators of disease biology and candidate tools for future precision-medicine strategies rather than validated predictive biomarkers for selecting patients most likely to benefit from high-dose intravenous vitamin C therapy.

**Figure 2:**
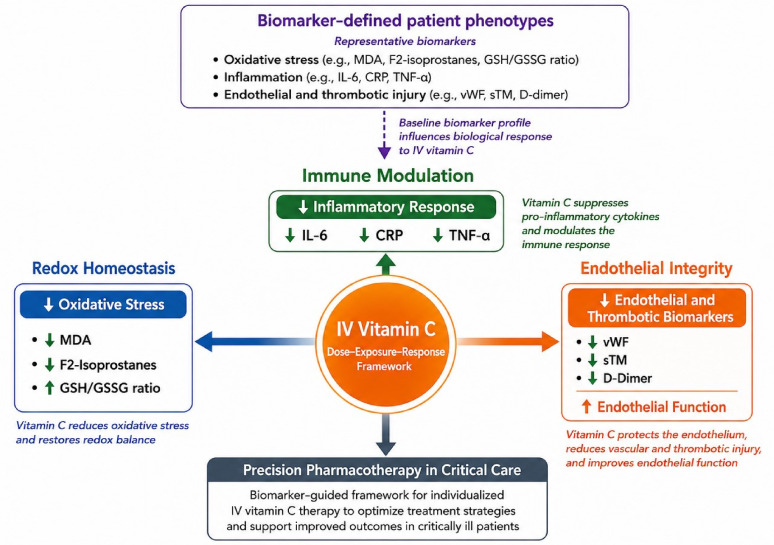
Precision redox–immune–endothelial framework for biomarker-guided intravenous vitamin C therapy in critical illness. The figure illustrates how baseline biomarker-defined patient phenotypes, reflecting oxidative stress, inflammation, and endothelial injury, may influence the biological response to intravenous vitamin C within a dose–exposure–response framework. Intravenous vitamin C is proposed to modulate interconnected redox, immune, and endothelial pathways, resulting in reduced oxidative stress, suppression of pro-inflammatory mediators, improved endothelial function, and supporting biomarker-guided precision pharmacotherapy in critically ill patients. Representative biomarkers are shown as examples of biological domains that may facilitate future patient stratification strategies and individualized therapeutic decision-making rather than as mandatory treatment-selection criteria. The magnitude of the biological and clinical response to intravenous vitamin C is expected to vary according to the patient’s baseline biomarker-defined phenotype, disease severity, and clinical context, consistent with the proposed dose–exposure–response framework. Abbreviations: CRP, C-reactive protein; D-dimer, fibrin degradation product D-dimer; GSH/GSSG ratio, ratio of reduced to oxidized glutathione; IL-6, interleukin-6; MDA, malondialdehyde; sTM, soluble thrombomodulin; TNF-α, tumor necrosis factor-α; vWF, von Willebrand factor.

Within this framework, high-dose intravenous vitamin C (HDIVC) can be interpreted as a mechanistically aligned therapy rather than a non-specific antioxidant. Its effects on redox signaling, inflammatory modulation, and endothelial function, including interactions with nitric oxide pathways, are consistent with the proposed endotypes (Figure 2) [73,90,102]. However, therapeutic efficacy appears contingent on both timing and phenotype, reinforcing the limitation of applying HDIVC uniformly across heterogeneous populations.

Advances in high-dimensional profiling, including multi-omics and immunometabolic signatures, provide the tools to translate these concepts into clinically actionable algorithms. Real-time phenotyping and adaptive modeling may enable continuous refinement of treatment strategies as patient data evolve [104].

Taken together, this approach establishes a translational bridge from molecular insight to bedside decision-making, supporting the design of enriched clinical trials and precision-based therapeutic algorithms that align interventions such as HDIVC with biologically defined patient subgroups [101,102,103,104,105,106,107,108].

### 7.1. Limitations and Future Directions

Despite strong mechanistic rationale, current evidence for intravenous vitamin C in critical illness remains limited by methodological and clinical heterogeneity. Major guidelines, including those from WHO, NIH, and the Surviving Sepsis Campaign, do not recommend routine use outside clinical trials [11,43,47,48,49]. Existing studies are constrained by small sample sizes, variable dosing strategies, inconsistent timing of administration, and lack of phenotype-based stratification, limiting both interpretability and generalizability [38,109,110].

From a clinical pharmacology perspective, these limitations reflect insufficient control of key determinants of therapeutic response: exposure (dose and administration strategy), timing relative to disease progression, and biological heterogeneity. Future trials should therefore prioritize standardized dosing protocols, pharmacokinetic endpoints, and biomarker-based patient selection to better define exposure–response relationships [111,112].

Integration of biomarker profiling—including inflammatory, oxidative, endothelial, and coagulation markers—with pharmacogenetic and multi-omics data offers a pathway toward more precise patient stratification and individualized dosing strategies [6,16,33,73,100,101,102,103,104,105,106]. In parallel, combination approaches with agents targeting complementary pathways (e.g., corticosteroids, thiamine, anticoagulants, vitamin D, zinc, N-acetylcysteine, melatonin) warrant systematic evaluation for synergistic effects [11,36,57,58,102].

Real-world data from registries and electronic health records may complement randomized trials by providing insight into safety, dosing patterns, and long-term outcomes across diverse populations [72,113,114,115]. Safety considerations remain essential, particularly regarding oxalate nephropathy, G6PD-related hemolysis, and infusion-related effects, emphasizing the need for appropriate screening and monitoring [35,71,74].

Collectively, these directions highlight the need to move beyond descriptive evaluation toward mechanistically informed, exposure-driven clinical investigation.

### 7.2. Integrative Model and Future Precision Trial Design

Future clinical trials of HDIVC should adopt a precision framework integrating biomarker-defined phenotypes and pharmacogenetic stratification. Inflammatory markers such as IL-6 may define hyperinflammatory endotypes, while composite endothelial indices (e.g., sTM and vWF) may identify patients with predominant vascular injury. Genetic variability in vitamin C transport (SLC23A1/2) may further refine patient selection by identifying individuals with differential intracellular uptake.

Combination strategies, including HDIVC with N-acetylcysteine or melatonin, may enhance redox modulation, mitochondrial function, and inflammasome regulation, thereby improving intermediate clinical endpoints. The integration of multi-omics approaches and machine learning can facilitate identification of responder profiles and support adaptive trial designs.

A comprehensive synthesis of current clinical evidence is presented in Table 8, linking clinical endpoints with mechanistic consistency across redox–immune–endothelial pathways. This integrative framework provides a foundation for designing trials that maximize therapeutic signal by aligning intervention, exposure, and patient phenotype.

## 8. Conclusions

Vitamin C exerts a range of biological effects extending beyond antioxidant activity, influencing immune responses, endothelial integrity, and vascular homeostasis. In critically ill patients, high-dose intravenous administration enables pharmacological exposure associated with measurable changes in inflammatory, oxidative, and endothelial biomarkers. High-dose intravenous administration enables pharmacologically active plasma concentrations associated with measurable pharmacodynamic effects, including modulation of inflammatory, oxidative, and endothelial biomarkers. However, biomarker modulation alone should not be interpreted as evidence of clinical efficacy, which ultimately requires demonstration of improvement in patient-important clinical outcomes [11,38,43,46,47,48,49,50,51,90,91,92,93,94,95,96,97,98].

However, the absence of consistent clinical benefit across randomized trials may reflect heterogeneity in dosing strategies, timing of administration, patient selection, and pharmacokinetic exposure. Equally, the possibility that HDIVC provides limited or no clinically meaningful benefit in some or all critically ill populations cannot currently be excluded. The available evidence supports further evaluation of approaches integrating pharmacokinetic principles with biomarker-guided stratification therefore represents a necessary step toward more effective and individualized use [6,16,33,73,100,101,102,103,104,105,106].

Within the redox–immune–endothelial framework, intravenous vitamin C may be conceptualized as a context-dependent pharmacological intervention rather than a universally applicable therapy. Identifying patients with modifiable redox imbalance, inflammatory activation, and endothelial dysfunction is central to optimizing its clinical role [101,102,103,104,105,106,107,108].

From a clinical pharmacology perspective, intravenous vitamin C should be understood as an exposure-dependent intervention, where dose, timing, and biological phenotype converge to determine therapeutic outcome. Future studies should prioritize moving beyond binary efficacy questions toward defining exposure–response relationships within biomarker-stratified patient populations, thereby evaluating the proposed framework prospectively rather than assuming its current clinical applicability.

### Clinical Implications for Precision Pharmacotherapy

– Intravenous administration is required to achieve pharmacologically active plasma concentrations
– Therapeutic effects are exposure-dependent and contingent on reaching millimolar levels
– Earlier administration, before irreversible inflammatory and endothelial injury becomes established, may represent an important determinant of therapeutic response.

However, the optimal therapeutic window remains to be defined and requires prospective validation in biomarker-guided randomized clinical trials.

– Biomarker-guided strategies using markers such as IL-6, CRP, D-dimer, and endothelial markers may enrich responder populations, although these approaches remain to be prospectively validated
– Genetic and metabolic variability (e.g., SLC23A1/2, G6PD) may influence both efficacy and safety

## Figures and Tables

**Table 1:** Comparison of Oral vs. Intravenous Vitamin C Administration.

Parameter	Oral Administration	Intravenous Administration	Clinical Pharmacology Interpretation	Reference
Bioavailability	Limited due to saturation of intestinal transporters	Nearly 100%; bypasses gastrointestinal absorption	Defines ceiling effect for oral dosing	[5]
Peak Plasma Concentration	~100–150 µmol/L	>10,000–20,000 µmol/L (10–20 mM)	IV enables transition from nutritional to pharmacological range	[5]
Pharmacodynamic Threshold	Typically not sufficient (<100 µM)	Achieved (>1–5 mM)	Required for redox and immune modulation	[5,32,33]
Absorption Mechanism	SVCT1 and SVCT2 (saturable active transport)	Direct systemic delivery; cellular uptake via SVCT transporters	Transporter saturation may limit intracellular exposure	[5,32]
Dose–Exposure Relationship	Non-linear (saturable)	Approximately linear at high doses	Determines predictability of pharmacological response	[5]
Therapeutic Use	Maintenance, prevention, mild deficiency	Acute conditions: sepsis, ARDS, severe COVID-19	Reflects the potential to achieve pharmacologically active plasma concentrations required for pharmacodynamic activity	[24,33]
Onset of Action	Slower; dependent on gastrointestinal absorption	Rapid; immediate systemic effect	Critical for early-phase intervention	[32]
Exposure Profile	Low, sustained	High peak ± sustained (depending on infusion strategy)	Peak vs. time-dependent pharmacodynamics	[5,32,33]
Advantages	Convenient, safe, low cost	Enables pharmacological concentrations and systemic effects	Route determines clinical applicability	[5]
Limitations	Dose restricted by absorption	Requires clinical supervision	Limits real-world implementation	[5]
Side Effects	Gastrointestinal upset, diarrhea	Rare: oxalate nephropathy, hemolysis in G6PD deficiency	Dose- and patient-dependent safety profile	[5]
Clinical Impact	Limited in critical illness	Potential modulation of inflammation and endothelial dysfunction	Exposure-dependent pharmacodynamic activity	[5]

Note: Intravenous vitamin C enables plasma concentrations several hundred-fold higher than those achievable with oral administration, exceeding the estimated pharmacodynamic threshold (>1–5 mM) required for modulation of redox-sensitive pathways. This distinction underpins the exposure-dependent transition from nutritional to pharmacological activity. Achievement of pharmacologically active plasma concentrations should not be interpreted as evidence of clinical efficacy, which ultimately depends on patient characteristics, disease stage, and demonstrated improvement in clinically relevant outcomes.

**Table 2:** Comparative Clinical Evidence Supporting Biomarker-Guided High-Dose Intravenous Vitamin C Therapy in Critical Illness.

Study (Year)	Population	Intervention	Proposed Mechanism(s)	Dose and Duration	Comparator	Primary Outcome(s)	Principal Findings	Strengths	Limitations
Labbani-Motlagh et al., 2022 [37]	Moderate–severe COVID-19	HDIVC	Antioxidant; immune modulation	12 g/day × 4 days	Placebo + standard care	SOFA score; 28-day mortality	No significant improvement in primary clinical outcomes despite pharmacologically relevant intravenous exposure	Randomized placebo-controlled study evaluating pharmacological-dose intravenous vitamin C.	No mortality benefit
Zhang et al., 2021 [38]	Severe/Critical COVID-19 (ICU)	HDIVC	Endothelial protection; immune modulation	12 g IV every 12 h × 7 days	Placebo	IMV-free days; 28-day mortality	Improved PaO_2_/FiO_2_ and reduced CRP; no significant mortality benefit	Objective assessment of oxygenation and inflammatory biomarkers	Primary endpoint not achieved
Zhao et al., 2021 [39]	Severe COVID-19	HDIVC	Antioxidant; immune modulation	Variable HDIVC regimens	Standard care	SOFA score; CRP; PaO_2_/FiO_2_	Improved inflammatory biomarkers and oxygenation	Demonstrated pharmacodynamic activity	Retrospective, non-randomized
CITRIS-ALI (Fowler et al.), 2019, randomized clinical trial [11]	Sepsis + ARDS	HDIVC	Endothelial protection; anti-inflammatory activity	50 mg/kg every 6 h × 4 days	Placebo	SOFA score; CRP; thrombomodulin	No significant improvement in the primary endpoint; secondary analyses suggested lower 28-day mortality and more ICU-free days, warranting further investigation.	Landmark RCT in sepsis	Primary endpoint not met; secondary outcome findings should be interpreted cautiously.
Xu et al., 2024 (Systematic review and Meta-analysis) [40]	Hospitalized COVID-19	Oral and/or IV vitamin C	Antioxidant; immune modulation	Variable regimens	Standard care	Mortality; ICU length of stay	No significant reduction in mortality or ICU length of stay; evidence of biological activity, but no consistent improvement in patient-important clinical outcomes.	Large pooled evidence	Substantial clinical heterogeneity; variability in route of administration, dosing regimens, and study quality
Adhikari et al., 2023 (LOVIT-COVID), Harmonized multicenter randomized clinical trials [41]	Critically ill patients with COVID-19	HDIVC	Redox modulation; endothelial protection, immune regulation	200 mg/kg/day × 4 days	Placebo	Organ support-free days; mortality; inflammatory biomarkers	No significant improvement in organ support-free days or mortality. Post hoc exploratory analyses suggested reduced inflammatory biomarker levels and lower vasopressor requirements among patients receiving earlier treatment; these findings require prospective validation.	Large multicenter harmonized randomized clinical trials with standardized protocol	Primary clinical endpoint not met; exploratory subgroup findings should be interpreted cautiously and require confirmation.
Luo et al., 2023 (systematic review and meta-analysis) [42]	Sepsis	IV vitamin C	Antioxidant; immunomodulatory	Approximately 6–24 g/day across included trials	Placebo + standard care	Short-term mortality, ICU length of stay, SOFA score, CRP	No significant improvement in short-term mortality, ICU length of stay, or SOFA score; reduced vasopressor duration and lower CRP levels; subgroup analyses suggested possible benefit in selected settings; these findings require cautious interpretation and prospective confirmation.	Comprehensive synthesis of randomized trials with predefined subgroup analyses	Considerable clinical and methodological heterogeneity; variability in dosing regimens, treatment duration, patient populations, and study quality.
Ahmed et al., 2026 (Systematic Review) [10]	Critically ill patients (2020–2025 studies)	HDIVC	Antioxidant; endothelial protection; immune modulation	Various regimens	Multiple RCTs and observational studies	Mortality; SOFA score; vasopressor use; ICU outcomes	No consistent mortality benefit; consistent signals of physiological and biomarker improvement; supports further evaluation in biomarker-guided precision trials	Most up-to-date evidence synthesis	High clinical and methodological heterogeneity

Notes: HDIVC: High-dose intravenous vitamin C; ROS: Reactive oxygen species; SOFA: Sequential Organ Failure Assessment; IMV: Invasive mechanical ventilation; PaO_2_/FiO_2_: arterial oxygen partial pressure/fraction of inspired oxygen ratio; CRP: C-reactive protein; IL-6: interleukin-6. Overall, current evidence consistently demonstrates biological and physiological activity of high-dose intravenous vitamin C. However, evidence of clinically meaningful benefit remains heterogeneous across randomized trials. Whether treatment response is influenced by pharmacokinetic exposure, timing of administration, patient phenotype, and disease stage remains a biologically plausible hypothesis that requires prospective validation.

**Table 3:** Intravenous Vitamin C Formulations, Dosing Regimens, and Pharmacokinetic Considerations Across Representative Clinical Studies.

Study (Author, Year)	Formulation	Dose	Duration	Patient Population	Key Outcomes	Notes
Fowler et al., 2019 [11]	Ascorbic acid solution	50 mg/kg every 6 h (12 g/day)	96 h (4 days)	Sepsis with ARDS	Primary endpoint not met; exploratory secondary analyses suggested potential clinical benefit	Buffered solution; infusion-related irritation rare
Zhang et al., 2021 [38]	Sodium ascorbate	12 g/day	7 days	Critically ill COVID-19	Improved oxygenation (PaO_2_/FiO_2_) and reduced inflammatory markers.	Sodium salt improved formulation stability
Fujii et al., 2020 (VITAMINS Trial) [62]	Ascorbic acid + thiamine + hydrocortisone	1.5 g IV every 6 h (6 g/day) + thiamine 200 mg every 12 h + hydrocortisone 50 mg every 6 h	Up to 7 days	Adults with septic shock (ICU)	No improvement in vasopressor-free days or mortality	Combination therapy well tolerated; outcomes similar to hydrocortisone alone
Marik et al., 2017 [36]	Ascorbic acid + thiamine	1.5 g IV every 6 h (6 g/day)	4 days	Sepsis with shock	Mortality reduction in observational study	Part of combination therapy
Kanji et al., 2026 [12]	Intravenous vitamin C	50 mg/kg every 6 h (12 g/day)	96 h (4 days)	Adults with sepsis (LOVIT PK substudy)	Substantial interindividual pharmacokinetic variability despite standardized dosing.	Supports future exposure-guided dosing strategies; vitamin C clearance was independently associated with renal function (CKD-EPI eGFR) and illness severity (APACHE II).

Note: Intravenous ascorbic acid formulations are typically adjusted to near-physiologic pH (≈5–7) using buffering agents to minimize infusion-related vein irritation. High concentrations increase solution osmolarity, which may contribute to local infusion discomfort. Rare adverse events—including oxalate nephropathy and hemolysis—require careful monitoring, particularly in patients with renal impairment or glucose-6-phosphate dehydrogenase (G6PD) deficiency. The optimal mode of administration (continuous infusion versus intermittent bolus dosing) remains under investigation. While intermittent bolus administration may achieve higher peak plasma concentrations, recent population pharmacokinetic evidence demonstrates that identical HDIVC dosing can result in substantial interindividual variability in systemic exposure, largely influenced by renal function and illness severity. These findings support an exposure-aware interpretation of clinical outcomes and further reinforce the rationale for biomarker-guided precision therapy. Abbreviations: ARDS, acute respiratory distress syndrome; G6PD, glucose-6-phosphate dehydrogenase; HDIVC, high-dose intravenous vitamin C; ICU, intensive care unit; LOVIT, Lessening Organ Dysfunction with Vitamin C Trial.

**Table 4:** Pharmacogenetic Factors Potentially Affecting the Efficacy of Intravenous Vitamin C.

Gene	Function/Polymorphism	Variant Example (rsID)	Possible Impact on Pharmacokinetics or Pharmacodynamics	Suggested Clinical Implication	Clinical Significance	References
*SLC23A1* (SVCT1)	Sodium-dependent vitamin C transporter 1; polymorphisms may affect intestinal absorption and cellular uptake	rs33972313	Altered plasma and tissue vitamin C concentrations, potentially influencing baseline status and antioxidant availability	↓ Plasma ascorbate levels; may require higher IV dosing to achieve target concentrations	May influence baseline vitamin C status and interindividual variability in response; relevance for dosing requires further investigation	[63,64]
*SLC23A2* (SVCT2)	Sodium-dependent vitamin C transporter 2; polymorphisms may affect intracellular distribution	rs6139591	Changes in intracellular vitamin C concentrations, modulating antioxidant capacity and redox balance	↓ Intracellular transport efficiency; reduced tissue saturation under oxidative stress	Possible impact on tissue response under oxidative stress; clinical implications not yet fully established	[59,60]
*GSTM1* (Glutathione S-transferase Mu 1)	Enzyme involved in detoxification and antioxidant metabolism; null polymorphism common	Null genotype	May alter glutathione-mediated antioxidant defense, influencing redox regulation and interaction with vitamin C metabolism	↓ Glutathione recycling; lower antioxidant defense, potential blunted response to vitamin C therapy	Polymorphism may modulate antioxidant response; clinical significance remains uncertain—more studies needed	[61,65]
G6PD (Glucose-6-phosphate dehydrogenase)	Key enzyme maintaining redox balance; deficiency impairs NADPH and glutathione recycling	c.376A→G (rs1050829)	Increased susceptibility to oxidative hemolysis under high oxidative load, including high-dose vitamin C exposure	↑ Risk of hemolysis; avoid IV doses > 10 g/day in G6PD-deficient patients	Elevated risk of hemolysis with high-dose IV vitamin C in G6PD-deficient patients; screening recommended prior to therapy	[36,66]

Note: This table summarizes pharmacogenetic factors that may influence the pharmacokinetics and pharmacodynamics of intravenous vitamin C. While variants in *SLC23A1* and *SLC23A2* have been associated with differences in vitamin C plasma and tissue concentrations, their impact on clinical efficacy during intravenous therapy remains unclear. The *GSTM1* polymorphism could modulate antioxidant defense, but current evidence is limited and primarily observational. In contrast, G6PD deficiency has a well-documented risk of oxidative hemolysis following high-dose vitamin C administration; screening before therapy is advisable. Overall, these findings highlight the potential for personalized approaches yet emphasize the need for further validation in clinical settings. Symbols: ↑ denotes an increase in the indicated parameter or risk; ↓ denotes a decrease or reduction in the indicated parameter, activity, or efficiency.

**Table 5:** Biomarkers Relevant for Monitoring and Stratification of Patients Receiving High-Dose Intravenous Vitamin C Therapy.

Biomarker	Association with Disease Outcomes	Role in Predicting Response to Vitamin C Therapy	Assay Type	Predictive Cut-off (if Known)	References
Interleukin-6 (IL-6)	Elevated levels correlate with severe inflammation and poor outcomes in COVID-19 and ARDS	Predictor of inflammatory status; reduction associated with vitamin C’s anti-inflammatory effects	ELISA	>100 pg/mL—hyperinflammatory phenotype	[90,91,92]
C-reactive Protein (CRP)	Marker of systemic inflammation; higher levels linked to worse clinical outcomes	Changes in CRP may reflect response to anti-inflammatory vitamin C treatment	Immunoturbidimetry	>150 mg/L—poor-outcome predictor	[93,94]
Malondialdehyde (MDA)	Product of lipid peroxidation; elevated in critical illness and ARDS	Decrease suggests reduced lipid peroxidation and oxidative damage	TBARS/LC-MS	>3.5 µmol/L—oxidative-stress phenotype	[95]
F2-Isoprostanes	Reliable biomarker of oxidative stress in vivo	Reduction indicates improved redox balance under vitamin C treatment	LC-MS/MS	>50 pg/mL—indicative of oxidative injury	[96]
Von Willebrand Factor (vWF)	Elevated in endothelial injury, associated with severity of sepsis and ARDS	Reduction may indicate endothelial repair during therapy	ELISA/Immunoassay	>250% activity—marker of endothelial activation	[97]
Soluble Thrombomodulin (sTM)	Marker of endothelial cell damage	Decrease may reflect improved endothelial integrity	ELISA	>7.5 ng/mL—endothelial-injury phenotype	[98]
D-dimer	Elevated levels associated with coagulopathy and worse outcomes in COVID-19 and sepsis	Reduction may reflect decreased inflammation and coagulation abnormalities	Immunoturbidimetry	>2.0 mg/L FEU—high risk of coagulopathy	[99]

Note: This table summarizes key inflammatory, oxidative stress, and endothelial biomarkers relevant to the pathophysiology of sepsis, ARDS, and severe COVID-19. Reported assay methods and threshold values reflect those most frequently applied in clinical and translational studies. Such standardized biomarker characterization facilitates patient stratification, monitoring of treatment response, and alignment with precision-guided therapeutic protocols involving intravenous vitamin C.

**Table 6:** Established Safety Monitoring Parameters Recommended Before and During High-Dose Intravenous Vitamin C Therapy.

Safety Parameter	Clinical Significance	Recommended Monitoring	Recommended Clinical Action	Key References
Glucose-6-phosphate dehydrogenase (G6PD) status	Identifies patients at increased risk of oxidative hemolysis during pharmacological-dose vitamin C therapy.	Quantitative G6PD screening before initiation of HDIVC, particularly when very high doses are planned or G6PD deficiency is suspected.	Avoid HDIVC or administer with caution in patients with confirmed G6PD deficiency; consider alternative treatment strategies when appropriate.	[75,79,80,81]
Renal function (serum creatinine, eGFR, urine output)	Identifies patients at increased risk of oxalate nephropathy and acute kidney injury associated with vitamin C metabolism.	Baseline renal assessment before treatment initiation and serial monitoring throughout therapy.	Individualize treatment according to renal function and monitor closely for early signs of oxalate nephropathy or acute kidney injury.	[75,76,77,78]
Urinary oxalate (when clinically indicated)	Detects excessive oxalate accumulation in patients at increased risk of oxalate nephropathy.	Measurement in selected patients with impaired renal function, prolonged HDIVC administration, or unexplained deterioration of renal function.	Facilitate early recognition of oxalate nephropathy and guide further nephrological evaluation when appropriate.	[77,78]
Laboratory plasma glucose	Pharmacological plasma vitamin C concentrations may produce spuriously elevated point-of-care glucose measurements, creating a risk of inappropriate or overly aggressive insulin administration and subsequent severe hypoglycemia.	Use laboratory-based plasma glucose measurements during and immediately after HDIVC infusion whenever accurate glycemic assessment is required.	Avoid reliance on susceptible point-of-care glucometers and prevent inappropriate insulin administration caused by falsely elevated glucose readings.	[83,84,85]
Iron overload/hereditary hemochromatosis	Identifies patients at increased risk of iron-catalyzed oxidative injury due to enhanced reduction of ferric (Fe^3+^) to ferrous (Fe^2+^) iron by vitamin C.	Ferritin and iron studies (e.g., transferrin saturation) when clinically indicated or when iron overload is suspected.	Individualize therapy and use HDIVC cautiously in patients with hereditary hemochromatosis or clinically significant iron overload.	[75,76,82,86]

Note: These safety-monitoring parameters complement the efficacy biomarkers presented in Table 5. Whereas Table 5 summarizes biomarkers reflecting pharmacodynamic response, inflammation, oxidative stress, endothelial injury, and coagulation abnormalities, the present table focuses on clinically established safety-monitoring measures recommended before and during high-dose intravenous vitamin C therapy. Together, these complementary monitoring strategies support individualized risk assessment, treatment optimization, and biomarker-guided precision pharmacotherapy in critically ill patients. Abbreviations: CPIC, Clinical Pharmacogenetics Implementation Consortium; eGFR, estimated glomerular filtration rate; Fe^3+^, ferric iron; Fe^2+^, ferrous iron; G6PD, glucose-6-phosphate dehydrogenase; HDIVC, high-dose intravenous vitamin C.

**Table 7:** Redox–Immune–Endothelial Axes in Precision Redox Medicine: Mechanisms, Biomarkers, and Targeted Interventions.

Axis	Key Mechanisms	Representative Biomarkers	Potential Clinical Endpoints	Redox-Targeted Interventions
Redox modulation	ROS/RNS scavenging, antioxidant recycling, glutathione regeneration	Malondialdehyde (MDA), F2-isoprostanes, GSH/GSSG ratio	↓ oxidative stress markers, ↓ multi-organ failure, improved SOFA	High-dose IV Vitamin C, N-acetylcysteine (NAC), Thiamine
Immune recalibration	IL-6 and NLRP3 inflammasome suppression, T-cell and cytokine modulation	IL-6, CRP, TNF-α, IL-1β	↓ cytokine storm, ↓ systemic inflammation, ↓ SOFA	IV Vitamin C ± Corticosteroids, Thiamine, Melatonin
Endothelial stabilization	Syndecan-1 preservation, ↓ sTM and vWF release, modulation of nitric oxide signaling	Syndecan-1, Soluble Thrombomodulin (sTM), von Willebrand factor (vWF), D-dimer	Improved PaO_2_/FiO_2_ ratio, ↓ vasopressor requirements, ↓ ICU stay duration	IV Vitamin C + Melatonin or NAC (combination therapy)

Note: This integrative framework aligns key molecular axes—redox, immune, and endothelial—with their corresponding biomarkers and therapeutic interventions. It serves as a translational bridge connecting molecular mechanisms to clinical decision-making in critically ill patients with sepsis, ARDS, or severe COVID-19. Emerging evidence also suggests that renal injury phenotypes may represent an additional dimension of future precision therapeutic algorithms. Symbols: ↓ denotes a decrease or reduction in the indicated parameter, biomarker level, risk, or clinical outcome.

**Table 8:** Comparative Evidence Supporting Biomarker-Guided High-Dose Intravenous Vitamin C Therapy in Critical Illness.

Clinical Endpoint	Overall Clinical Evidence	Certainty of Evidence (GRADE)	Biological Rationale for Precision Therapy
IL-6/CRP ↓	Evidence suggests benefit	Moderate	Supported by translational and preclinical evidence demonstrating cytokine suppression, attenuation of oxidative stress, and immune modulation.
PaO_2_/FiO_2_ ↑	Evidence suggests benefit	Moderate	Consistent improvement following early administration; mechanistically linked to endothelial protection, attenuation of pulmonary oxidative injury, and improved oxygenation.
Acute kidney injury (AKI)	Possible benefit	Low	Emerging randomized evidence suggests reduced incidence of AKI and renal replacement therapy through preservation of renal microvascular perfusion and attenuation of oxidative renal injury; confirmation in adequately powered biomarker-guided RCTs is required.
Vasopressor requirement ↓	Evidence suggests benefit	Moderate	Biologically plausible through restoration of endogenous catecholamine synthesis via the dopamine β-hydroxylase cofactor effect together with improved vascular responsiveness.
ICU length of stay ↓	Possible benefit	Low–moderate	Reported in several randomized trials and meta-analyses; however, considerable heterogeneity persists regarding patient selection, dosing strategies, timing of therapy, and disease severity.
Mortality ↓	Inconsistent evidence	Low	Current evidence remains inconclusive. Future phenotype-stratified and biomarker-guided randomized controlled trials are required to identify patients most likely to benefit.
Endothelial injury markers (sTM, vWF, D-dimer) ↓	Evidence suggests benefit	Moderate	Mechanistically consistent with endothelial glycocalyx preservation, reduced endothelial activation, improved vascular integrity, and attenuation of thrombo-inflammatory responses.

Note: Summary trends are synthesized from the randomized clinical trials, systematic reviews, and meta-analyses summarized in Table 2 together with emerging evidence incorporated during manuscript revision. Certainty of evidence was qualitatively appraised according to GRADE principles considering study design, consistency of findings, directness of evidence, and biological plausibility. Biological rationale reflects concordance between observed clinical outcomes and the proposed redox–immune–endothelial mechanisms underlying biomarker-guided precision therapy. The potential benefit for acute kidney injury (AKI) is supported by recent pilot randomized evidence and should currently be regarded as hypothesis-generating pending confirmation in adequately powered biomarker-guided clinical trials. Biological rationale and pharmacodynamic biomarker modulation provide mechanistic support for intravenous vitamin C therapy but should not be interpreted as evidence of clinical efficacy. Patient-important outcomes remain the primary standard for evaluating therapeutic benefit. Symbols: ↑ denotes an increase or improvement in the indicated clinical parameter or outcome; ↓ denotes a decrease or reduction in the indicated biomarker level, clinical parameter, risk, or outcome.

## Data Availability

No new data were created or analyzed in this study.

## Abbreviations:

Abbreviation	Definition
ACE2	Angiotensin-Converting Enzyme 2
APACHE II	Acute Physiology and Chronic Health Evaluation II
ARDS	Acute Respiratory Distress Syndrome
CKD-EPI	Chronic Kidney Disease Epidemiology Collaboration
COVID-19	Coronavirus Disease 2019
CRP	C-Reactive Protein
eGFR	Estimated Glomerular Filtration Rate
G6PD	Glucose-6-Phosphate Dehydrogenase
HDIVC	High-Dose Intravenous Vitamin C
ICU	Intensive Care Unit
IL-6	Interleukin-6
IMV	Invasive Mechanical Ventilation
NLRP3	NOD-, LRR-, and Pyrin Domain-Containing Protein 3 Inflammasome

## References

[1] Beltrán-García J., Osca-Verdegal R., Pallardó F.V., Ferreres J., Rodríguez M., Mulet S., Sanchis-Gomar F., Carbonell N., García-Giménez J. (2020). L Oxidative stress and inflammation in COVID-19-associated sepsis: The potential role of anti-oxidant therapy in avoiding disease progression. Antioxidants.

[2] Cheng R.Z., Kogan M., Davis D. (2020). Ascorbate as Prophylaxis and Therapy for COVID-19-Update from Shanghai and U.S. Medical Institutions. Glob. Adv. Health Med..

[3] JamaliMoghadamSiahkali S., Zarezade B., Koolaji S., SeyedAlinaghi S.A., Zendehdel A., Tabarestani M., Sekhavati Moghadam E., Abbasian L., Dehghan Manshadi S.A., Salehi M. (2021). Safety and effectiveness of high-dose vitamin C in patients with COVID-19: A randomized open-label clinical trial. Eur. J. Med. Res..

[4] Alissa A., Alrashed M.A., Alshaya A.I., Sulaiman K.A., Alharbi S. (2024). Reevaluating vitamin C in sepsis and septic shock: A potential benefit in severe cases?. Front. Med..

[5] Padayatty S.J., Sun H., Wang Y., Riordan H.D., Hewitt S.M., Katz A., Wesley R.A., Levine M. (2004). Vitamin C pharmacokinetics: Implications for oral and intravenous use. Ann. Intern. Med..

[6] Lykkesfeldt J., Carr A.C., Tveden-Nyborg P. (2025). Pharmacological Reviews The pharmacology of vitamin C. Pharmacol. Rev..

[7] Cameron E., Pauling L. (1976). Supplemental ascorbate in the supportive treatment of cancer. Proc. Natl. Acad. Sci. USA.

[8] Creagan E.T., Moertel C.G., O’Fallon J.R., Schutt A.J., O’Connell M.J., Rubin J., Frytak S. (1979). Failure of high-dose vitamin C (ascorbic acid) therapy to benefit patients with advanced cancer. N. Engl. J. Med..

[9] Moertel C.G., Fleming T.R., Creagan E.T., Rubin J., O’Connell M.J., Ames J.F., McDonald M.T., Loprinzi C.L., Jolles J.W., Tschetter L.K. (1985). High-dose vitamin C versus placebo in the treatment of patients with advanced cancer who have had no prior chemotherapy. N. Engl. J. Med..

[10] Ahmed R., Shetty S., Aziz Khan M.Q., Kalsoom I., Qasim S., Khan S.B., Abdullah S. (2026). Intravenous Vitamin C in Severe Sepsis: A Systematic Review of Evidence from 2020 to 2025. Cureus.

[11] Fowler A.A., Truwit J.D., Hite R.D., Morris P.E., DeWilde C., Priday A., Fisher B., Thacker L.R., Natarajan R., Brophy D.F. (2019). Effect of vitamin C infusion on organ failure and biomarkers of inflammation and vascular injury in patients with sepsis and severe acute respiratory failure: The CITRIS-ALI Randomized Clinical Trial. JAMA.

[12] Kanji S., Hernández-Mitre M.P., Blain H., Adhikari N.K.J., Lamontagne F., Battista M.C., Ménard J., Sprague S., Masse M.-H., Roberts J.A. (2026). Population pharmacokinetics of intravenous vitamin C in adults with sepsis: A sub-study of the LOVIT trial. Clin. Pharmacokinet..

[13] Dragoi C.M., Diaconu C.C., Nicolae A.C., Dumitrescu I.B. (2024). Redox Homeostasis and Molecular Biomarkers in Precision Therapy for Cardiovascular Diseases. Antioxidants.

[14] Singer M., Deutschman C.S., Seymour C.W., Shankar-Hari M., Annane D., Bauer M., Bellomo R., Bernard G.R., Chiche J.-D., Coopersmith C.M. (2016). The Third International Consensus Definitions for Sepsis and Septic Shock (Sepsis-3). JAMA.

[15] Ranieri V.M., Rubenfeld G.D., Thompson B.T., Ferguson N.D., Caldwell E., Fan E., Camporota L., Slutsky A.S., Antonelli M., Anzueto A. (2012). Acute respiratory distress syndrome: The Berlin Definition. JAMA.

[16] Wilson R.B., Liang Y., Kaushal D., Carr A. (2024). Molecular Pharmacology of Vitamin C and Relevance to Health and Obesity—A Narrative Review. Int. J. Mol. Sci..

[17] Zheng H., Xu Y., Liehn E.A., Rusu M. (2024). Vitamin C as scavenger of reactive oxygen species during healing after myocardial infarction. Int. J. Mol. Sci..

[18] Calder P.C., Kreider R.B. (2025). Enhanced Vitamin C Delivery: A Systematic Literature Review Assessing the Efficacy and Safety of Alternative Supplement Forms in Healthy Adults. Nutrients.

[19] Kozlov A.V., Javadov S. (2024). Cellular ROS and Antioxidants: Physiological and Pathological Role. Antioxidants.

[20] Sharma Y., Sumanadasa S., Shahi R., Woodman R., Mangoni A.A., Bihari S., Thompson C. (2024). Efficacy and safety of vitamin C supplementation in the treatment of community-acquired pneumonia: A systematic review and meta-analysis with trial sequential analysis. Sci. Rep..

[21] Hosseinpour A., Daneshzad E., Abdi R., Shokoofeh D., Mostafa Z. (2023). The Association Between Antioxidants and COVID-19 Outcomes: A Systematic Review on Observational Studies. Biol. Trace Elem. Res..

[22] Carr A.C., Shaw G.M., Fowler A.A., Natarajan R. (2015). Ascorbate-dependent vasopressor synthesis: A rationale for vitamin C administration in severe sepsis and septic shock?. Crit. Care..

[23] Koçak Tufan Z., Kayaaslan B., Mer M. (2021). COVID-19 and Sepsis. Turk. J. Med. Sci..

[24] Nabzdyk C.S., Bittner E.A., Nabzdyk C.S., Bittner E.A. (2018). Vitamin C in the critically ill-indications and controversies. World J. Crit. Care Med..

[25] Zheng M., Williams E.P., Karki R., Malireddi R.K.S., Banoth B., Burton A., Webby R., Channappanavar R., Jonsson C.B., Kanneganti T.-D. (2020). Impaired NLRP3 inflammasome activation/pyroptosis leads to robust inflammatory cell death via caspase-8/RIPK3 during coronavirus infection. J. Biol. Chem..

[26] Barnett K.C., Li S., Liang K., Ting J.P.Y. (2023). A 360° view of the inflammasome: Mechanisms of activation, cell death, and diseases. Cell.

[27] Chen W., Gullett J.M., Tweedell R.E., Kanneganti T.D. (2023). Innate immune inflammatory cell death: PANoptosis and PANoptosomes in host defense and disease. Eur. J. Immunol..

[28] Lan J., Ge J., Yu J., Shan S., Zhou H., Fan S. (2020). Structure of the SARS-CoV-2 spike receptor-binding domain bound to the ACE2 receptor. Nature.

[29] Jackson C.B., Farzan M., Chen B., Choe H. (2022). Mechanisms of SARS-CoV-2 entry into cells. Nat. Rev. Mol. Cell Biol..

[30] Grishin A.M., Dolgova N.V., Landreth S., Fisette O., Pickering I.J., George G.N., Falzarano D., Cygler M. (2022). Disulfide Bonds Play a Critical Role in the Structure and Function of the Receptor-binding Domain of the SARS-CoV-2 Spike Antigen. J. Mol. Biol..

[31] Pasini A.M.F., Stranieri C., Cominacini L., Mozzini C. (2021). Potential Role of Antioxidant and Anti-Inflammatory Therapies to Prevent Severe SARS-CoV-2 Complications. Antioxidants.

[32] Harrison F.E., May J.M. (2009). Vitamin C function in the brain: Vital role of the ascorbate transporter SVCT2. Free Radic. Biol. Med..

[33] Flora S.D.E., Balansky R., Maestra S.L.A. (2021). Antioxidants and COVID-19. J. Prev. Med. Hyg..

[34] Fowler A.A., Syed A.A., Knowlson S., Sculthorpe R., Farthing D., DeWilde C., Farthing C.A., Larus T.L., Martin E., Brophy D.F. (2014). Phase I safety trial of intravenous ascorbic acid in patients with severe sepsis. J. Transl. Med..

[35] Fontana F., Cazzato S., Giovanella S., Ballestri M., Leonelli M., Mori G., Alfano G., Ligabue G., Magistroni R., Cenacchi G. (2020). Oxalate nephropathy caused by excessive vitamin C administration in 2 patients with COVID-19. Kidney Int. Rep..

[36] Marik P.E., Khangoora V., Rivera R., Hooper M.H., Catravas J. (2017). Hydrocortisone, vitamin C and thiamine for the treatment of severe sepsis and septic shock: A retrospective before-after study. Chest.

[37] Labbani-Motlagh Z., Amini S., Aliannejad R., Sadeghi A., Shafiee G., Heshmat R., Jafary M., Talaschian M., Akhtari M., Jamshidi A. (2022). High-dose Intravenous Vitamin C in Early Stages of Severe Acute Respiratory Syndrome Coronavirus 2 Infection: A Double-blind, Randomized, Controlled Clinical Trial. J. Res. Pharm. Prac..

[38] Zhang J., Rao X., Li Y., Zhu Y., Liu F., Guo G., Luo G., Meng Z., de Backer D., Xiang H. (2021). Pilot trial of high-dose vitamin C in critically ill COVID-19 patients. Ann. Intensive Care.

[39] Zhao B., Ling Y., Li J., Peng Y., Huang J., Wang Y., Qu H., Gao Y., Li Y., Hu B. (2021). Beneficial aspects of high dose intravenous vitamin C on patients with COVID-19 pneumonia in severe condition: A retrospective case series. Ann. Palliat. Med..

[40] Xu W., Wang P., Wan J., Tan Y., Liu Y., Chen Q., Zheng Y., Yu X., Fan S., Cuyubamba Dominguez J.L. (2024). Effect of vitamin C supplementation on outcomes in patients with COVID-19: A systematic review and meta-analysis. Front. Nutr..

[41] Adhikari N.K.J., Hashmi M., Tirupakuzhi Vijayaraghavan B.K., LOVIT-COVID Investigators, on behalf of the Canadian Critical Care Trials Group, The REMAP-CAP Investigators (2023). Intravenous vitamin C for patients hospitalized with COVID-19: Two harmonized randomized clinical trials. JAMA.

[42] Luo Q., Zhu W., Zhang F., Zhu Y., Kuang Y., Shao X., Guo X., Ning B. (2023). The effect of vitamin C in adults with sepsis: A meta-analysis of randomized controlled trials. Front. Med..

[43] Zeng Y., Liu Z., Xu F., Tang Z. (2023). Intravenous high-dose vitamin C monotherapy for sepsis and septic shock. A meta-analysis of randomized controlled trials. Medicine.

[44] Tamanna S., Kim C.-M., Lee Y.M., Seo J.-W., Kim D.Y., Yun N.R., Kim D.-M. (2025). Serum melatonin as a potential biomarker for COVID-19 severity. Sci. Rep..

[45] Abdo M., Kohaf N., Hammad M.A., Ping C.C. (2023). The Role of Oral Ascorbic Acid Administration in Combination with IV N-acetylcysteine in Delaying Inflammatory Cascade in Sepsis: A Case Report. Cureus.

[46] Kwak S.G., Choo Y.J., Chang M.C. (2022). The effectiveness of high-dose intravenous vitamin C for patients with coronavirus disease 2019: A systematic review and meta-analysis. Complement. Ther. Med..

[47] Wen C., Li Y., Hu Q., Liu H., Xu X., Muhan L. (2023). IV Vitamin C in Sepsis: A Latest Systematic Review and Meta-Analysis. Int. J. Clin. Pract..

[48] Rosengrave P., Spencer E., Williman J., Mehrtens J., Morgan S., Doyle T., Van Der Heyden K., Morris A., Shaw G., Carr A.C. (2022). Intravenous Vitamin C Administration to Patients with Septic Shock: A Pilot Randomised Controlled Trial. Crit. Care.

[49] Corrao S., Raspanti M., Agugliaro F., Gervasi F., Di Bernardo F., Natoli G., Argano C. (2024). Safety of High-Dose Vitamin C in Non-Intensive Care Hospitalized Patients with COVID-19: An Open-Label Clinical Study. J. Clin. Med..

[50] Gonzalez-Vazquez S.A., Gomez-Ramirez E.E., Gonzalez-Lopez L., Gamez-Nava J.I., Peraza-Zaldivar J.A., Santiago-Garcia A.P., Ramirez-Villafaña M., Gonzalez-Ponce F., Gomez-Camarena J.J., Saldaña-Cruz A.M. (2024). Intravenous Vitamin C as an Add-on Therapy for the Treatment of Sepsis in an Intensive Care Unit: A Prospective Cohort Study. Medicina.

[51] Tariq M.A., Amin H., Ali U. (2022). Meta-analysis assessing the effectiveness of intravenous vitamin C in patients with sepsis and septic shock. Acute Crit. Care.

[52] Zhang Y., Ding S., Li C., Wang Y., Chen Z.H.E., Wang Z. (2017). Effects of N-acetylcysteine treatment in acute respiratory distress syndrome: A meta-analysis. Exp. Ther. Med..

[53] Lu X., Ma Y., He J., Li Y., Zhu H. (2019). N-acetylcysteine for adults with acute respiratory distress syndrome: A meta-analysis of randomized controlled trials. Hong Kong J. Emerg. Med..

[54] González-Guzmán D., Andrade-Castellanos C.A., Ponce-Gallegos M.A., Mesina-Estarrón I., Mora-Almanza J.G., Ruelas-Moreno H.E., Rodríguez-González D., Eguia-Ortega O., Colunga-Lozano L.E. (2025). N-Acetyl-Cysteine in Intensive Care Unit Patients with Acute Respiratory Distress Syndrome due to COVID-19: A Retrospective Cohort Study. J. Intensive Care Med..

[55] Qin J., Wang G., Han D. (2025). Benefits of melatonin on mortality in severe-to-critical COVID-19 patients: A systematic review and meta-analysis of randomized controlled trials. Clinics.

[56] Huan S., Hong L., Lee Z., Chin L., Chih L., Lai C. (2022). Efficacy of melatonin in the treatment of patients with COVID-19: A systematic review and meta-analysis of randomized controlled trials. J. Med. Virol..

[57] Palli E., Makris D., Papanikolaou J., Garoufalis G., Tsilioni I., Zygoulis P. (2017). The impact of N-acetylcysteine and ascorbic acid in contrast-induced nephropathy in critical care patients: An open-label randomized controlled study. Crit. Care.

[58] Borges L., Gennari-felipe M., Dias B.B., Hatanaka E. (2022). Melatonin, Zinc, and Vitamin C: Potential Adjuvant Treatment for COVID-19 Patients. Front. Nutr..

[59] Liang W.J., Johnson D., Jarvis S.M. (2001). Vitamin C transport systems in human cells. Mol. Membr. Biol..

[60] May J.M. (2011). The SLC23 Family of Ascorbate Transporters: Ensuring That You Get and Keep Your Daily Dose of Vitamin C. Br. J. Pharmacol..

[61] Block G., Shaikh N., Jensen C.D., Volberg V., Holland N. (2011). Serum vitamin C and other biomarkers differ by genotype of phase II enzyme genes GSTM1 and GSTT1. Am. J. Clin. Nutr..

[62] Fujii T., Luethi N., Young P.J. (2020). Effect of Vitamin C, Hydrocortisone, and Thiamine vs. Hydrocortisone Alone on Time Alive and Free of Vasopressor Support Among Patients with Septic Shock. The VITAMINS Randomized Clinical Trial. JAMA.

[63] Michels A.J., Hagen T.M., Frei B. (2013). Human Genetic Variation Influences Vitamin C Homeostasis by Altering Vitamin C Transport and Antioxidant Enzyme Function. Annu. Rev. Nutr..

[64] Timpson N.J., Forouhi N.G., Brion M.-J., Harbord R.M., Cook D.G., Johnson P., McConnachie A., Morris R.W., Rodriguez S., Luan J. (2010). Genetic Variation at the SLC23A1 Locus Is Associated with Circulating Levels of L-Ascorbic Acid (Vitamin C): Evidence from 5 Independent Studies with Over 15,000 Participants. Am. J. Clin. Nutr..

[65] Josephy P.D. (2010). Genetic Variations in Human Glutathione Transferase Enzymes: Significance for Pharmacology and Toxicology. Int. J. Mol. Sci..

[66] Juneja D., Jain R., Nasa P. (2022). Vitamin C-induced Hemolysis: Meta-summary and Review of Literature. Indian J. Crit. Care Med..

[67] Linowiecka K., Foksiński M., Brożyna A.A. (2020). Vitamin C transporters and their implications in carcinogenesis. Nutrients.

[68] Lykkesfeldt J., Poulsen H.E. (2010). Is Vitamin C Supplementation Beneficial? Lessons Learned from Randomised Controlled Trials. Br. J. Nutr..

[69] May J.M., Qu Z.C. (2005). Transport and intracellular accumulation of vitamin C in endothelial cells: Relevance to collagen synthesis. Arch. Biochem. Biophys..

[70] Townsend D.M., Tew K.D. (2003). The Role of Glutathione-S-Transferase in Anti-Cancer Drug Resistance. Oncogene.

[71] Luzzatto L., Ally M., Notaro R. (2020). Glucose-6-phosphate dehydrogenase deficiency. Blood.

[72] Corrigan-Curay J., Sacks L., Woodcock J. (2018). Real-world evidence and real-world data for evaluating drug safety and effectiveness. JAMA.

[73] Wick K.D., McAuley D.F., Levitt J.E., Beitler J.R., Annane D., Riviello E.D., Calfee C.S., Matthay M.A. (2021). Promises and challenges of personalized medicine to guide ARDS therapy. Crit. Care.

[74] Cappellini M.D., Fiorelli G. (2008). Glucose-6-phosphate dehydrogenase deficiency. Lancet.

[75] Alangari A., Arif J., Al Qureshah F., Alkhodairy F. (2026). Clinical benefits and risks of high-dose intravenous vitamin C: A systematic review. J. Med. Life.

[76] PDQ Integrative, Alternative, and Complementary Therapies Editorial Board PDQ^®^ Intravenous Vitamin C. Bethesda (MD): National Cancer Institute (US); Updated 2025 May 13. https://www.cancer.gov/about-cancer/treatment/cam/hp/vitamin-c-pdq.

[77] Zou G., Lim J., Rosenberg A.Z. (2025). Oxalate nephropathy from high-dose intravenous vitamin C in a patient with multiple myeloma. J. Nephrol..

[78] Rosenstock J.L., Joab T.M.J., DeVita M.V., Yang Y., Sharma P.D., Bijol V. (2022). Oxalate nephropathy: A review. Clin. Kidney J..

[79] Marik P.E. (2019). Is intravenous vitamin C contraindicated in patients with G6PD deficiency?. Crit. Care.

[80] Honore P.M., Kugener L., Redant S., Attou R., De Bels D. (2020). Harm of IV High-Dose Vitamin C Therapy in Adult Patients: A Scoping Review. Crit. Care.

[81] Gammal R.S., Pirmohamed M., Somogyi A.A., Morris S.A., Formea C.M., Elchynski A.L., Oshikoya K.A., McLeod H.L., Haidar C.E., Whirl-Carrillo M. (2023). Expanded Clinical Pharmacogenetics Implementation Consortium guideline for medication use in the context of G6PD genotype. Clin. Pharmacol. Ther..

[82] Gerster H. (1999). High-dose vitamin C: A risk for persons with high iron stores?. Int. J. Vitam. Nutr. Res..

[83] Carr A.C., Cook J. (2018). Intravenous vitamin C for cancer therapy–identifying the current gaps in our knowledge. Front. Physiol..

[84] Rosengrave P.C., Wohlrab C., Spencer E., Williman J., Shaw G., Carr A.C. (2023). Effect of intravenous vitamin C on arterial blood gas analyser and Accu-Chek point-of-care glucose monitoring in critically ill patients. Crit. Care Resusc..

[85] Howell A.P., Parrett J.L., Malcom D.R. (2021). Impact of High-Dose Intravenous Vitamin C for Treatment of Sepsis on Point-of-Care Blood Glucose Readings. J. Diabetes Sci. Technol..

[86] Zhao H., Fu W., Yang X., Zhang W., Wu S., Ma J., Zhang T., Yao H., Zhang Z. (2025). High-dose vitamin C: A promising anti-tumor agent, insight from mechanisms, clinical research, and challenges. Genes Dis..

[87] Nauman G., Gray J.C., Parkinson R., Levine M., Paller C.J. (2018). Systematic review of intravenous ascorbate in cancer clinical trials. Antioxidants.

[88] Gao P., Shen Y., Wu P., Lv W. (2024). Ascorbic acid-induced warfarin resistance after breast cancer surgery: A case report and literature review. Front. Pharmacol..

[89] Thien T.T., Truong P.V., Ratna O., Ha H.-A. (2024). Heliyon Comparative efficacy of antioxidant therapies for sepsis and septic shock in the intensive care unit: A frequentist network. Heliyon.

[90] Yin J.X., Agbana Y.L., Sun Z.S., Fei S.W., Zhao H.Q., Zhou X.N. (2023). Increased interleukin—6 is associated with long COVID-19: A systematic review and meta-analysis. Infect. Dis. Poverty..

[91] Gholizadeh M., Abdul S., Saeedy G., Abdi A. (2021). Vitamin C reduces interleukin-6 plasma concentration: A systematic review and meta-analysis of randomized clinical trials. Clin. Nutr. Open Sci..

[92] Grudlewska-Buda K., Wiktorczyk-Kapischke N., Budzyńska A., Kwiecińska-Piróg J., Przekwas J., Kijewska A., Sabiniarz D., Gospodarek-Komkowska E., Skowron K. (2022). The Variable Nature of Vitamin C—Does It Help When Dealing with Coronavirus?. Antioxidants.

[93] Villoteau A., Asfar M., Otekpo M., Loison J., Gautier J., Annweiler C. (2021). Elevated C-reactive protein in early COVID-19 predicts worse survival among hospitalized geriatric patients. PLoS ONE.

[94] Balafar M., Mahmoodpoor A., Arjmandi H., Khelejani A.M., Soleimanpour H. (2024). High-Dose Vitamin C in the Treatment of COVID-19 Patients in Intensive Care Unit; A Letter to the Editor. Arch. Acad. Emerg. Med..

[95] Kochlik B., Grune T., Weber D. (2017). New findings of oxidative stress biomarkers in nutritional research. Curr. Opin. Clin. Nutr. Metab. Care.

[96] Dietrich M., Block G., Benowitz N.L., Morrow J.D., Hudes M., Jacob P. (2003). Vitamin C Supplementation Decreases Oxidative Stress Biomarker F2-Isoprostanes in Plasma of Nonsmokers Exposed to Environmental Tobacco Smoke. Nutr. Cancer.

[97] Vassiliou A.G., Kotanidou A., Dimopoulou I., Orfanos S.E. (2020). Endothelial Damage in Acute Respiratory Distress Syndrome. Int. J. Mol. Sci..

[98] Liu Z., Li Y., Zhao Q., Kang Y. (2023). Association and predictive value of soluble thrombomodulin with mortality in patients with acute respiratory distress syndrome: Systematic review and meta-analysis. Ann. Transl. Med..

[99] Informasi M., Kedokteran I., Kesehatan D.A.N. (2024). Ascorbic Acid Supplementation on D-dimer Levels in COVID-19 Patients. J. Kedokt..

[100] Olczak-Pruc M., Swieczkowski D., Ladny J.R., Pruc M., Juarez-Vela R., Rafique Z., Peacock F.W., Szarpak L. (2022). Vitamin C Supplementation for the Treatment of COVID-19: A Systematic Review and Meta-Analysis. Nutrients.

[101] Van Nynatten L.R., Bokhary D., Wong M.Y.S., Wang J., Fero H., McChesney C., Fiorini K., Blake L., Fraser D.D., Slessarev M. (2025). Predictive Enrichment Using Biomarkers in Studies of Critically-Ill Patients with Sepsis: A Systematic Review. Crit. Care.

[102] Xiao Y., Gong F., Zhang L., Gui C. (2025). Vitamin C for sepsis: From mechanisms to individualized therapy. Front. Med..

[103] Paul S.N., Nessel I., Puthucheary Z., Henson S. (2026). Sepsis and the immunometabolic inflammatory response. npj Metab. Health Dis..

[104] Jin X., Shen H., Zhou P., Yang J., Yang S., Ni H., Yu Y., Zhang Z. (2025). Research Progress on Sepsis Diagnosis and Monitoring Based on Omics Technologies: A Review. Diagnostics.

[105] Ma W., Tang S., Yao P., Zhou T., Niu Q., Liu P., Wang X., Zhang Y., Li H., Chen J. (2025). Advances in acute respiratory distress syndrome: Focusing on heterogeneity, pathophysiology, and therapeutic strategies. Signal Transduct. Target. Ther..

[106] Moskowitz A., Andersen L.W., Huang D.T., Berg K.M., Grossestreuer A.V., Marik P.E., Sherwin R.L., Hou P.C., Becker L.B., Cocchi M.N. (2018). Ascorbic acid, corticosteroids, and thiamine in sepsis: A review of the biologic rationale and the present state of clinical evaluation. Crit. Care.

[107] Hwang S.Y., Ryoo S.M., Park J.E., Jo Y.H., Jang D.H., Suh G.J., Kim T., Kim Y.-J., Kim S., Cho H. (2020). Combination therapy of vitamin C and thiamine for septic shock: A multi-centre, double-blinded randomized, controlled study. Intensive Care Med..

[108] Li Z., Zhang X., Wu Y., Xie C., Liu C., He X., Yang H., Wang Y., Chen L. (2023). Hydrocortisone, vitamin C, and thiamine may not improve the outcome of patients with sepsis or septic shock: A systematic review and meta-analysis. Emerg. Crit. Care Med..

[109] Juneja D., Nasa P., Jain R. (2022). Current role of high dose vitamin C in sepsis management: A concise review. World J. Crit. Care Med..

[110] Gavrielatou E., Xourgia E., Xixi N.A., Mantelou A.G., Ischaki E., Kanavou A., Zervakis D., Routsi C., Kotanidou A., Siempos I.I. (2022). Effect of Vitamin C on Clinical Outcomes of Critically Ill Patients with COVID-19: An Observational Study and Subsequent Meta-Analysis. Front. Med..

[111] Yong S., Liu S., Zhang P., Dong L., Wei Q. (2024). The effects of vitamin C supplementation in the critically ill patients outcomes: A systematic review and meta-analysis of randomized controlled trials. Medicine.

[112] Feng F., Yang H., Yang W., Li M. (2021). Effect of vitamin C in critically ill patients with sepsis and septic shock: A meta-analysis. Sci. Prog..

[113] Sherman R.E., Anderson S.A., Dal Pan G.J., Gray G.W., Gross T., Hunter N.L., LaVange L., Marinac-Dabic D., Marks P.W., Robb M.A. (2016). Real-world evidence—What is it and what can it tell us?. N. Engl. J. Med..

[114] Parums D. (2025). A Review of the Importance and Relevance of Real-World Data and Real-World Evidence. Med. Sci. Monit..

[115] Costa V., Custodio M.G., Gefen E., Fregni F. (2025). The relevance of the real-world evidence in research, clinical and regulatory decision making. Front. Public Health.

